# Reoperation after the failure of Wang procedure on pectus excavatum: Wung procedure + Wenlin procedure

**DOI:** 10.1093/jscr/rjac499

**Published:** 2022-10-30

**Authors:** Wenlin Wang, Weiguang Long, Yang Liu, Bin Cai, Juan Luo

**Affiliations:** Department of Chest Wall Surgery, Guangdong Second Provincial General Hospital, Guangzhou, China; Department of Chest Wall Surgery, Guangdong Second Provincial General Hospital, Guangzhou, China; Department of Chest Wall Surgery, Guangdong Second Provincial General Hospital, Guangzhou, China; Department of Chest Wall Surgery, Guangdong Second Provincial General Hospital, Guangzhou, China; Department of Chest Wall Surgery, Guangdong Second Provincial General Hospital, Guangzhou, China

## Abstract

Wang procedure for pectus excavatum is a simple and safe surgery. However, improper operation may lead to failure of the surgery. We received a 7-year-old male patient with pectus excavatum who failed Wang procedure. We used the combination of Wung procedure and Wenlin procedure to correct the deformity and achieved satisfactory results.

## INTRODUCTION

Wang procedure is a new technique for pectus excavatum introduced in recent years, which can correct the depression and obtain satisfactory results [1]. However, it has special technical requirements for operation. If it is not operated properly, it may lead to the failure of the operation. Recently, we received a 7-year-old child with pectus excavatum who had experienced a failed Wang procedure. We performed Wung procedure [2] combined with Wenlin procedure [3, 4] for him and obtained a satisfactory result.

## CASE REPORT

**Figure 1:**
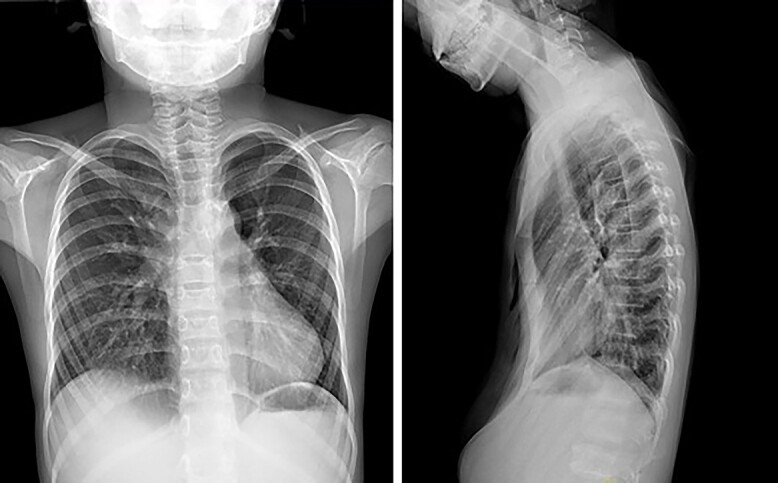
Chest X-ray examination before the first operation.

**Figure 2:**
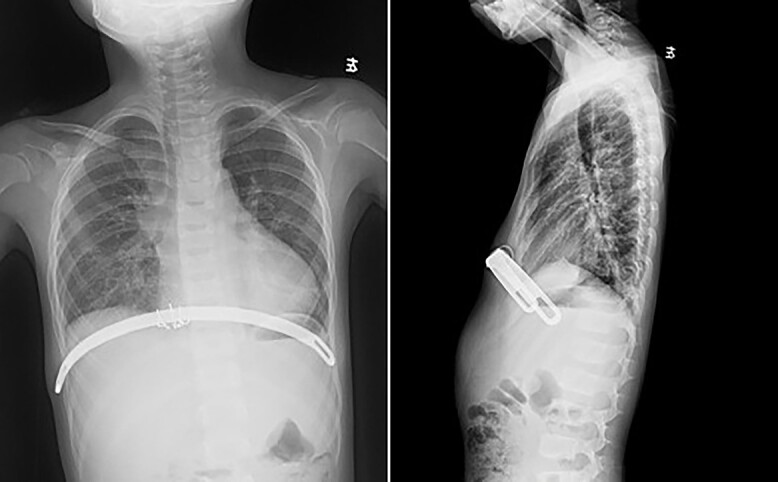
Chest X-ray examination after the first operation.

**Figure 3:**
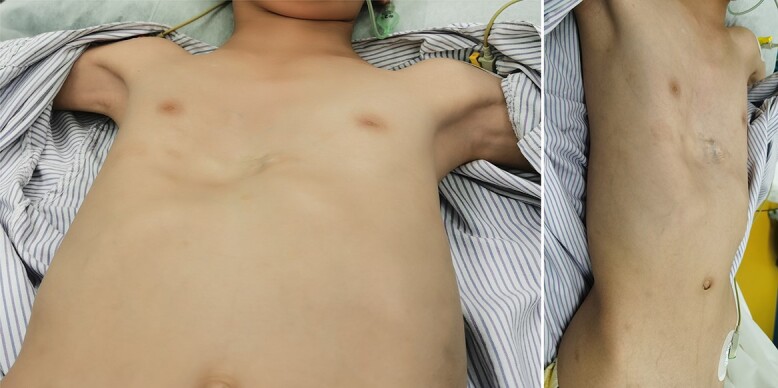
Appearance of chest wall before reoperation.

**Figure 4:**
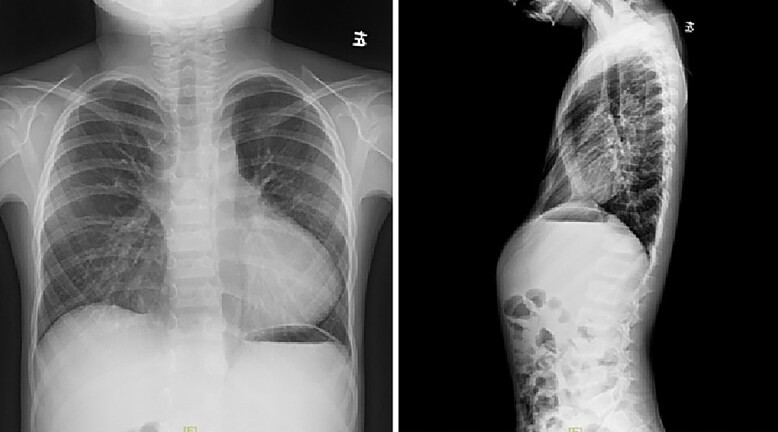
Chest X-ray examination before reoperation.

**Figure 5:**
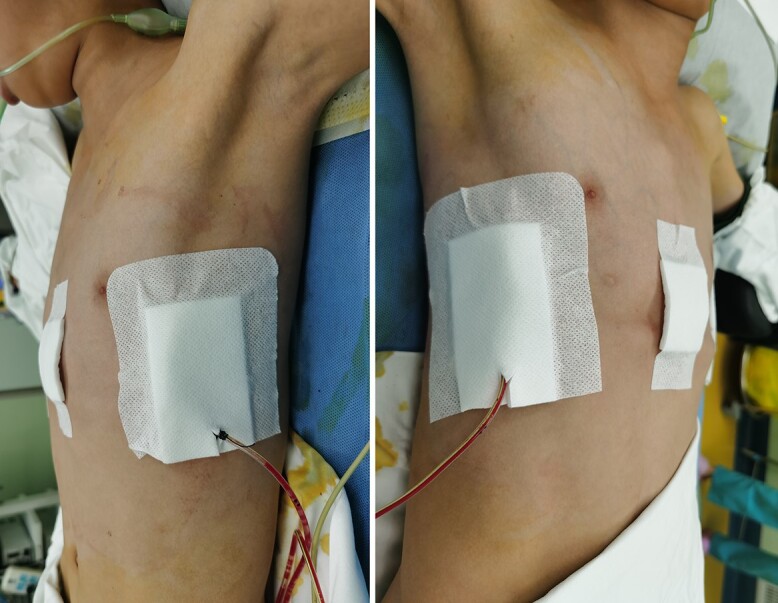
Appearance of chest wall after reoperation.

**Figure 6:**
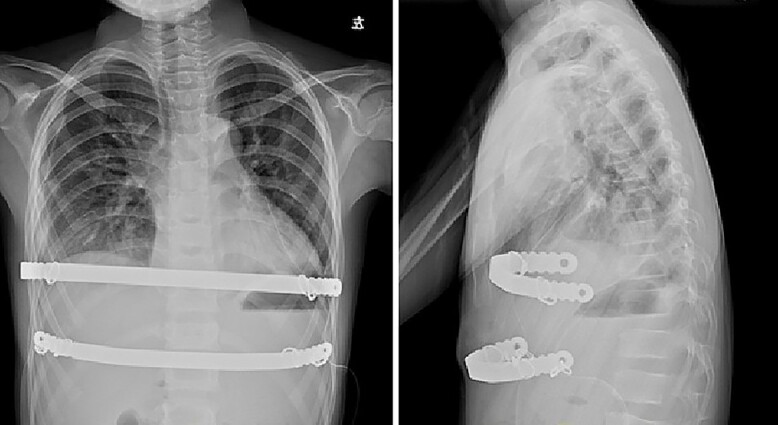
Chest X-ray examination after reoperation.

The patient was a 7-year-old boy. Two years ago, he underwent surgical treatment for pectus excavatum in a local hospital. At that time, Wang procedure was used and a steel bar was implanted. After the operation, the steel bar was displaced and the depression recurred (Figs 1 and 2), so the steel bar had to be taken out 1 year after the operation. After removal, his anterior chest wall still had obvious depression, and there was a tendency to aggravate. In order to completely correct the deformity, the patient was recently admitted to our hospital for surgery. Preoperative physical examination showed that the anterior chest wall was asymmetrically depressed, and the deepest depression was located in the right chest wall (Fig. 3). There was a scar in the middle of the anterior chest wall (Fig. 3). Preoperative imaging examination showed that the anterior chest wall was depressed, the heart was obviously compressed and moved to the left (Fig. 4). The operation was performed under general anesthesia. Supine position was adopted, with abduction of both upper limbs. Two incisions were made on both lateral chest wall respectively, which were located at the deepest plane of the depression. The muscles were dissected to expose the ribs in the incision. The depression was eliminated by Wung procedure, which was performed as follows [2]: A tunnel at the deepest plane of the depression was made. A special guider was inserted directly through the gap between the sternum and the heart, and then a steel bar introducing tube was connected with the guider. After the introducing tube was pulled into the body by the guider, a special steel bar was pulled by the introducing tube to the bottom of the depression. After the steel bar was rotated, the depression was supported, but the left chest wall began to be protrusive. In order to make the anterior chest wall more beautiful, Wenlin procedure was performed next [3, 4]. An incision at the median scar was made, and a tunnel was built from the median incision to the incision on both sides, which was located between the bone structures and the chest wall muscles. The second steel bar was inserted into the tunnel. After the protrusive part of the anterior chest wall was pressed with the middle part of the steel bar, both sides of the bar was fixed to the ribs on the lateral chest wall. Drainage tubes were placed in the thoracic cavity on both sides, the incision was closed, and the operation was completed (Fig. 5). No complications occurred during the operation. The total operation time was 50 min and the intraoperative bleeding volume was 5 ml. Postoperative X-ray examination showed that the positions of the steel bars were satisfactory and the shape of the chest wall was basically normal (Fig. 6). The patient was discharged 5 days after operation. Follow up for 1 month showed no change in thoracic shape and no discomfort.

## DISCUSSION

Pectus excavatum is the most common thoracic deformity, and Nuss procedure has been regarded as the standard operation for the treatment of this deformity [5, 6]. However, this procedure is not perfect. In order to eliminate the disadvantages of Nuss procedure, a new operation has appeared in the clinic, which is Wang procedure [1]. Wang procedure is to correct the depression from its surface. Because the whole operation can be completed under direct vision, it has proved to be a safe and simple operation. However, it has special requirements for operational details. If there are problems with the operation itself, it is easy to lead to the failure of the operation.

This patient is an asymmetric pectus excavatum. This pathological feature is not suitable for Wang procedure. Because the depression could not be completely lifted by the steel wires, the operation was easy to fail.

For the reoperation of patients with pectus excavatum surgery failure, Wang procedure was originally an ideal choice [7]. However, since the first operation was Wang procedure, the second operation is not suitable for Wang procedure. Considering the disadvantages of standard Nuss procedure, we chose Wung procedure for this patient [2]. Wung procedure is actually a modified Nuss procedure, which is an ideal surgical method because it has been greatly improved in many technical details [2]. For reoperation patients, this operation is always safer and better than other operations.

Wenlin procedure is specially designed for pectus carinatum [3, 4]. In later practice, it is often used for various protrusive deformities [8–12]. For this patient, after the completion of Wung procedure, because the surface of the anterior chest wall did not meet the expected standard, we used Wenlin procedure to correct it. The essence of this kind of surgery is template plastic surgery [13], therefore, the appearance of the chest wall can be improved to the greatest extent.

In short, Wang procedure is a simple and safe operation for the treatment of pectus excavatum, but if there are problems in the operation, it may also lead to failure. For the reoperation after the failure of pectus excavatum surgery, the combination of Wung procedure and Wenlin procedure can be selected for correction. Such a combination can achieve the most satisfactory results.

## CONFLICT OF INTEREST STATEMENT

None declared.

## FUNDING

None.
